# Misattribution of musical arousal increases sexual attraction towards opposite-sex faces in females

**DOI:** 10.1371/journal.pone.0183531

**Published:** 2017-09-11

**Authors:** Manuela M. Marin, Raphaela Schober, Bruno Gingras, Helmut Leder

**Affiliations:** 1 Department of Basic Psychological Research and Research Methods, University of Vienna, Vienna, Austria; 2 Department of Psychology, University of Innsbruck, Innrain, Innsbruck, Austria; Boston Children's Hospital / Harvard Medical School, UNITED STATES

## Abstract

Several theories about the origins of music have emphasized its biological and social functions, including in courtship. Music may act as a courtship display due to its capacity to vary in complexity and emotional content. Support for music’s reproductive function comes from the recent finding that only women in the fertile phase of the reproductive cycle prefer composers of complex melodies to composers of simple ones as short-term sexual partners, which is also in line with the ovulatory shift hypothesis. However, the precise mechanisms by which music may influence sexual attraction are unknown, specifically how music may interact with visual attractiveness cues and affect perception and behaviour in both genders. Using a crossmodal priming paradigm, we examined whether listening to music influences ratings of facial attractiveness and dating desirability of opposite-sex faces. We also tested whether misattribution of arousal or pleasantness underlies these effects, and explored whether sex differences and menstrual cycle phase may be moderators. Our sample comprised 64 women in the fertile or infertile phase (no hormonal contraception use) and 32 men, carefully matched for mood, relationship status, and musical preferences. Musical primes (25 s) varied in arousal and pleasantness, and targets were photos of faces with neutral expressions (2 s). Group-wise analyses indicated that women, but not men, gave significantly higher ratings of facial attractiveness and dating desirability after having listened to music than in the silent control condition. High-arousing, complex music yielded the largest effects, suggesting that music may affect human courtship behaviour through induced arousal, which calls for further studies on the mechanisms by which music affects sexual attraction in real-life social contexts.

## Introduction

Music is part of every human culture [1], and humans have engaged with music in many ways for at least 40.000 years [2]. Music can take forms such as song, dance and instrumental music, and each has played a pervasive role in societies because of their effects on cognition, emotion and behaviour [3]. However, the origins of music remain elusive, and a wide spectrum of theories, including adaptationist and non-adaptationist proposals, has sought to account for music’s roots [4]. Adaptationist theories emphasized the strong biological and social function of music, such as playing a role in courtship [5], social group cohesion [6] and mother-infant bonding [7], but the precise mechanisms through which music achieves such an impact on people’s social lives remain largely unknown. Here, we examine whether musical sound plays a role in sexual attraction towards opposite-sex faces, and if so, whether misattribution of arousal and excitation, rather than pleasantness transfer, underlies this phenomenon. We also explored possible effects of gender and menstrual cycle phase, which may moderate music’s effect on sexual attraction.

Focusing on sexual selection and attraction in particular, Charles Darwin [5] proposed in *The descent of man* (1871) that music, like birdsong, is a biological adaptation and has a reproductive function. Music may act as a courtship display because it can vary in complexity and emotional content, and musical ability may serve as an “honest” signal of fine motor skills [8] or advanced cognitive abilities [9]. In other words, engaging with music may be a signal indicating biological fitness (i.e., “good genes”). Importantly, Darwin [5] (p. 875) did not view music as a sexually dimorphic trait, which is in line with the fact that both men and women produce and enjoy music. Darwin also proposed that music and language have a common origin, a theory which is referred to as the “musical protolanguage” hypothesis.

Behavioural paradigms in real-life social interactions provided some empirical support for Darwin’s assertion [10, 11], but these studies focused on the effects of visual displays of musical ability on sexual attraction, rather than on musical sound. Further, these studies did not control for women’s menstrual cycle phase, hormonal contraception use and musical training. Focusing on the impact of music experienced as sound, Charlton et al. [12] tested whether women in the fertile phase of the cycle show enhanced preference for complex music and reported a general preference for complex music, but no effect of cycle phase. They further argued that their paradigm involving ratings of music might not have been suitable to demonstrate a preference for composers of complex music. In a large follow-up online study, Charlton [9] recently showed that only women in the fertile phase of the reproductive cycle preferred composers of complex melodies, rather than composers of simple melodies, as short-term sexual partners. However, Charlton [9] used artificial musical stimuli and did not test male participants. Nevertheless, anthropological evidence complements Charlton’s findings [9], indicating that in several tribes around the globe playing an instrument is used to attract a mate [13, 14]. It is yet unclear whether conscious appreciation of the musical skills of a potential partner leads to mate choice. An alternative view is that affective, rather than cognitive, mechanisms may as well lead to attraction, or that cognitive and affective mechanisms may interact with each other.

In general, it is a challenging task to provide convincing empirical evidence for or against Darwin’s theory, especially since current biological and psychological research has provided conflicting results [8, 9, 12, 15, 16, 17]. In our view, the task at hand is to reflect on the exact mechanisms through which music (song, dance and instrumental music) and/or musical ability may have affected sexual attraction in a reproductive, social context in which music was performed and experienced in groups. Because the human face is a salient signal during mate choice [18], we regard the systematic study of how music affects face processing in the context of sexual attraction as critical. Specifically, we studied whether music can affect facial attractiveness and dating behaviour.

This new avenue of research of the direct influence of music on the processing of faces is also pertinent to research on the crossmodal transfer of emotions. Music can alter the perception of emotional expressions of human faces [19], and there is some indication that sounds [20] and background music [21], but see [22] for counter evidence, can change the perceived facial attractiveness. However, previous studies did not differentiate between effects of musical arousal and pleasantness, two core dimensions of emotions [23], which also underlie musical emotions [24, 25]. Differential effects of arousal and pleasantness on face processing are very likely, considering that such effects have been shown when processing environmental scenes, with high-arousing music leading to higher arousal ratings of pictures in the absence of an analogous transfer of pleasantness from music to pictures [26]. This needs to be taken into account when testing the underlying mechanisms of Darwin’s theory on the origins of music, because musical arousal, but not pleasantness, is a strong correlate of musical complexity [25].

Crossmodal transfer of arousal is germane to the study of the misattributed arousal effect. Arousal induced by one source may influence people’s evaluations of other potential sources of arousal [27]. Misattribution of arousal has been reported in the context of romantic and sexual attraction [28, 29, 30, 31] and can be explained by excitation transfer between two successive stimuli (or events) [32]. For instance, White et al. [31] showed that listening to either a comedy tape or a tape corresponding to a mutilation scene increases the liking of an attractive female confederate and decreases the liking of an unattractive female confederate, in comparison to a control condition involving a non-arousing tape (textbook excerpt), suggesting that both positively- and negatively-valenced arousal can be misattributed. More recently, Meston and Frohlich [30] approached men and women before and after a roller-coaster ride and showed that people reported higher degrees of sexual attractiveness and dating desirability towards a photograph of an opposite-sex individual of average attractiveness after exiting the ride, in comparison to people tested before entering the ride.

To the best of our knowledge, music-induced excitation transfer on sexual attraction has not been investigated yet, although effects of affective pictures and films on music appreciation were previously reported [33]. Clearly, one would also expect to observe an excitation transfer effect of musical arousal (and/or complexity) on sexual attraction, especially since musical arousal prior to the availability of sound recording was always experienced in a social context in which both musicians and listeners were present at the same time and place [34]. If music plays a role in courtship behavior, and if misattribution of arousal is a rather general effect, this basic psychological mechanism may be an additional indirect pathway of how music experienced as sound can enhance sexual attraction among humans. This mechanism may come into play even if the person under question is neither the creator nor the performer of that music, consciously trying to impress the desired partner. However, it is likely that this effect is enhanced if the person under question is explicitly identified as the composer or performer of the music.

The ovulatory shift hypothesis predicts that women prefer markers of genetic fitness when evaluating men for short-term mates during peak fertility, whereas traits that are valued in long-term mates, such as warmth and faithfulness, are not sensitive to cycle shifts [35]. This suggests that women evolved to pursue mixed mating strategies, in which, depending on the social context, either short- or long-term mateships were sought [36]. Another difficulty of providing evidence for or against Darwin’s theory of sexual selection for music lies in current discrepancies shown by meta-analyses regarding the ovulatory shift hypothesis [37, 38, 39]. Indeed, there is an ongoing discussion about the efficiency of different methods used to determine fertility [40]. Charlton et al. [12] did not report a significant effect of fertility (assessed by ovulation tests) on musical preferences using a within-subjects design, whereas his more recent large-scale online study [9] used a between-subjects design to show that fertility, as assessed from the self-reported typical cycle length, played a role in short-term preference for composers of complex melodies.

In the present study, we introduced a musical priming paradigm to study how music may affect sexual attraction [5] by testing whether and how the perception of a musical stimulus varying in arousal (complexity) and pleasantness affects sexual attraction. We used ecologically-valid musical stimuli as primes and opposite-sex faces of average attractiveness as targets, and considered several participant-specific background variables, such as mood prior to the experiment, musical background, liking of musical primes as well as relationship status. Specifically, we sought to investigate how music affects the perception of facial attractiveness and dating desirability, thus combining two common measures of sexual attraction [30]. We addressed these questions in three groups of participants (women in the fertile phase, women in the infertile phase and men) and tested a concrete set of hypotheses: (1) Musical priming, compared to a control condition, increases perceived facial attractiveness and dating desirability in all three groups; (2) high-arousing (i.e., more complex music) music leads to higher ratings of facial attractiveness and dating desirability than low-arousing music; (3) musical pleasantness has no effect on facial attractiveness and dating desirability; (4) both men and women show effects of music on sexual attraction; (5) women in the fertile phase of the reproductive cycle show stronger effects of musical priming than women in the infertile phase.

## Materials and methods

### Participants

A total of 157 male and female participants, mostly psychology students at the University of Vienna, were tested. All participants were non-musicians (i.e., less than three years of formal musical training) and not musically active at the time of the experiment. Women confirmed that they were not taking hormonal contraceptives, and were neither pregnant nor breast-feeding. Twenty-nine participants had to be excluded, either due to technical problems during the experiment (*n* = 11), self-reported homo- or bisexual orientations (*n* = 11), or extreme mood states prior to the experiment (*n* = 7) (i.e., scoring 3 interquartile ranges (IQRs) below the first quartile, which suggest extreme negative mood, fatigue and/or disquietude in comparison to other participants in each of the three groups). Furthermore, women reporting an irregular cycle, as well as women who were on days 1–5 of their cycle (*n* = 16), were excluded to avoid any potential effects of menstruation on ratings.

After the experiment, the remaining 72 women were contacted to report the onset of their next menses. This information, together with the information on the onset of the last menses before the experiment, enabled the calculation of the actual cycle length. The fertile window of each woman was estimated by using an ovulation calculator (http://www.umrechnung.org/eisprung-bestimmen-berechnen/zykluseisprungkalender-fruchtbare-tage.htm), which is based on medical data and probability values for conception risk obtained by Wilcox et al. [41]. Forty women were assigned to the group of high conception risk (fertile phase of the cycle) and 32 women to the group of low conception risk (infertile phase). These groups were carefully matched with 40 men on average measures of several background variables that potentially affected responses to the musical primes (see Tables 1 and 2), yielding a final sample size of 96 participants (32 men, 32 women in the fertile phase and 32 women in the infertile phase). Importantly, participants included in the final analysis were selected by the first author before the experimental data was analysed and no changes were made thereafter.

**Table 1:** Participant characteristics I. Summary in terms of groups’ mean age, the three MBDF scores, years of musical training, role of music in life, and liking of 19^th^-century piano solo music.

Group	Age	Mood pos.-neg.	Alertness/fatigue	Quietude/Disquietude	Yrs. musical training	Role of music in life	Liking of piano solo music
Fertile women							
*M*	24.1	18.09	15.75	16.91	.91	5.25	5.72
*SD*	4.1	1.25	3.14	1.97	1.09	1.63	1.17
Infertile women							
*M*	23.4	17.63	14.69	16.09	.63	5.56	5.88
*SD*	4.3	1.84	3.13	2.48	1.04	1.19	1.29
Men							
*M*	24.4	18.06	16.16	17.19	.72	5.66	5.53
*SD*	4.4	1.54	2.71	2.13	1.05	1.18	1.05
K-W Tests							
*H*	1.09	1.11	3.82	3.95	1.45	.72	2.89
*p*	.580	.574	.148	.138	.485	.697	.236

*Note*. *M* = mean, *SD* = standard deviation, K-W = Kruskal-Wallis, *H* = test statistic of the Kruskal-Wallis test, *p* = calculated probability Yrs. = years, pos. = positive, neg. = negative. All degrees of freedom are 2. Role of music in life and liking of piano music were assessed on 7-point scales. *N* = 32 in each group. All variables were used to match groups.

**Table 2:** Participant characteristics II. Summary of groups’ musical listening behaviour and the reported interest in a one-night stand and long-term relationship.

Group	Listening to classical music	Active music listening	Passive music listening	Going to concerts	One-night stand	Long-term relationship
Fertile women						
*M*	3.94	4.38	5.38	3.41	3.91	3.69
*SD*	1.88	1.77	1.96	1.88	1.96	1.64
Infertile women						
*M*	4.09	4.94	5.94	3.47	4.88	4.66
*SD*	1.63	1.65	1.34	1.78	2.06	1.38
Men						
*M*	3.19	4.59	5.50	2.81	4.69	4.38
*SD*	1.53	1.72	1.48	1.60	2.13	1.81
K-W Tests						
*H*	4.81	1.55	2.08	2.50	4.70	6.66
*p*	.091	.461	.354	.287	.096	.036

*Note*. *M* = mean, *SD* = standard deviation, K-W = Kruskal-Wallis, *H* = test statistic of the Kruskal-Wallis test, *p* = calculated probability, all degrees of freedom are 2. All ratings were given on 7-point scales. *N* = 32 in each group. All variables, except for “one-night stand” and “long-term relationship”, were used to match groups.

To estimate the minimum required number of participants, an a priori statistical power analysis was performed using the program G*Power 3.1 [42]. Men and women were analysed separately because the visual targets differed between the two groups. We planned to test our hypotheses within the framework of mixed-design ANOVA. Meston and Frohlich [30] reported a moderate effect size for effects of roller-coaster excitation transfer on attractiveness ratings and dating desirability, thus we ran our analyses assuming medium effect sizes for within-subject comparisons.

For hypothesis 1, the power analysis with a medium effect size of .25 (Cohen’s *f*), an alpha of .05, a correlation among repeated measures of .70, and a desired power of .80 yielded a minimum sample size of 21 participants for the within-subject effect in the group of men. A similar analysis involving the two groups of women yielded a minimum total sample size of 22. Identical results were obtained for hypothesis 2. Hypothesis 5 referred to the group difference between women in the fertile and infertile phase, and a power analysis with the same settings as above yielded a minimum total sample size of 110 participants. Given that we applied several exclusion criteria, such as no hormonal contraceptive use and less than three years of musical training, it was not possible to recruit such a large sample among university students.

### Materials

Forty professionally photographed faces (twenty male) varying in attractiveness (excluding very unattractive and attractive faces) were selected from the data set used in Schacht et al. [43]. Faces (630 x 819 pixels) were standardized and shown in frontal view and gaze direction, with a neutral expression, and on a grey background. For both groups of women, the 20 male faces were shown in the critical trials of the priming experiment. Seventeen out of the 20 female faces were used in the distractor trials. For men, the presented sets of faces were reversed and female faces were shown in the critical trials. The set of female photographs comprised the following images (original numbers from [43]: 6, 8, 11, 13, 22, 23, 24, 27, 36, 38, 45, 47, 51, 68, 70, 80, 81, 90, 93, and 117. The set of male photographs comprised the following images: 3, 4, 35, 40, 52, 55, 57, 66, 71, 77, 79, 94, 95, 100, 107, 111, 118, 119, 122, and 125. The selected female (*M* = 3.71, *SD* = 1.00) and male (*M* = 3.72, *SD* = 1.03) faces were of average attractiveness (7-point scale with 1 = very attractive, 4 = neither-nor, and 7 = very non-attractive), see [43].

Eighty excerpts of 19^th^-century piano solo music were chosen from the stimulus set described in Marin and Leder [25]. This musical style was selected because it is mostly unfamiliar to participants, and because we previously showed that music in this style can be used to prime the emotional processing of environmental scenes [26]. The excerpts (25 s) were selected to differ in their capacity to induce felt arousal (low vs. high) and pleasantness (unpleasant vs. pleasant) in the listener and grouped into one of four categories labelled “low-arousing pleasant”, “low-arousing unpleasant”, “high-arousing pleasant” or “high-arousing unpleasant” based on subjective ratings obtained in [25]. Seventeen excerpts that could not be clearly assigned to one of these categories were used for the distractor trials.

Marin and Leder [25] showed that compression rate of music files is a significant predictor of subjective complexity. We thus obtained an objective measure of musical complexity to compare the complexity of low- and high-arousing excerpts, demonstrating the close association between arousal and complexity in music. All excerpts in waveform format were converted into FLAC (Free Lossless Audio Codec) format using Audacity 2.0.6 and the following settings: bit depth = 16 bit, level = 5. The two low-arousing (*M* = 1375660.83, *SD* = 152363.88) and the two high-arousing categories (*M* = 1651303.00, *SD* = 185530.41) were aggregated, and a Mann-Whitney Test on the file sizes in bytes revealed a highly significant difference with a large effect size, *U* = 209.00, *z* = -5.69, *p* < .001, *r* = -.64, indicating that low-arousing stimuli were much simpler than high-arousing ones.

### Procedure

The study protocol was approved by the local ethics committee at the University of Vienna (approval number 00100). The study was advertised (among a pool of non-musicians and women who were not taking hormonal contraceptives) as investigating the evolutionary background of facial attractiveness, and music was not specifically mentioned to avoid selection bias. Participants chose between course credits and a small monetary compensation of 10 Euro.

The experiment took place in a quiet room with constant lighting conditions. The experiment was implemented in Matlab R2011a (The MathWorks Inc., Natick, USA). The music was played through an external soundcard (E-MU audio interface 0204/USB) and Sennheiser HD 380 Pro headphones at a fixed loudness level. Faces were presented on a 19 inch screen (Iiyama, ProLite B1906S) placed approximately 70 cm away from the sitting participant.

Prior to the experiment, participants completed a short demographic questionnaire and version A of the three-dimensional mood questionnaire (MDBF A) by Steyer et al. [44]. Participants were also tested for visual acuity and colour blindness. Then, they were randomly assigned to one of the two blocks (control vs. priming) and rating orders (attractiveness—dating desirability vs. dating desirability—attractiveness). The orders of the blocks and ratings were counterbalanced across participants in each group. Participants were instructed to give spontaneous ratings. For each participant, the rating order was the same in both blocks. Participants performed one practice trial in each block to familiarize themselves with the procedure.

In the control condition, the presentation of each face was preceded by a message which read “The next trial will follow soon” that was displayed in the centre of the screen for 5 s, after which either a target or distractor face was presented for 2 s on a black background. The first of the two 7-point rating scales then appeared on the screen and participants gave their rating by mouse click without any time constraints. The instruction for facial attractiveness ratings read “Please report the perceived sexual attractiveness of the face” and the scale ranged from (1) “very unattractive” to (7) “very attractive”. The instruction for ratings of dating desirability read “Please report whether you would like to date this person” and participants could indicate their choice ranging between (1) “No, by no means” and (7) “Yes, by all means”. After both ratings were entered, the next trial was announced. All 37 faces (target faces and foils) were randomly presented. The musical primes were not rated for their affective content in order to avoid demand characteristics and due to constraints regarding the acceptable length of the study.

In the musical priming block, the 20 opposite-sex faces were repeated four times and randomly combined with one musical excerpt of each emotion quadrant, yielding 80 trials. Seventeen distractor trials (to prevent demand characteristics) were added by combining same-sex faces with musical excerpts that could not be clearly assigned to one of the emotion quadrants, see [25]. All trials were randomly presented and again a message reading “The next trial will follow soon” was displayed for 5 s prior to the beginning of the following trial. Participants were asked to look at a small white cross presented in the middle of the screen while listening to the musical prime (25 s) in order not to miss the onset of the presentation of the face (2 s) after the end of the musical excerpt. As in the control condition, participants rated each face for facial attractiveness and dating desirability.

After the experiment, participants filled in a self-developed short questionnaire. Among other things, questions referred to participants’ musical background (musical preference, listening behaviour, and musical training), their liking for the presented music in the experiment (i.e., 19^th^-century piano solo music), and their willingness to have a one night-stand or enter a long-term relationship with the most attractive persons shown in the experiment. Women also provided detailed information about their menstrual cycle. Three additional standardized questionnaires were administered but not evaluated. The experimental session lasted around 90 minutes. Participants were paid (or assigned course credit), debriefed and thanked. Women were also informed that they might be contacted again to offer information about the onset of their next menstruation.

### Statistical analysis

The data was analysed using IBM Statistics SPSS 21. In the case of violations of sphericity, Greenhouse-Geisser corrections were performed. Effect sizes are reported whenever appropriate. We followed Cohen’s [45] suggestions for interpreting effect sizes reported as eta squared or partial eta squared, with small = .01, medium = .06 and large = .14 (but see [46] for limitations of this interpretation for within-subject comparisons). Confidence intervals (CI) for effect sizes of between-subjects comparisons were computed in SPSS with a script downloaded from http://core.ecu.edu/psyc/wuenschk/StatHelp/StatHelp.htm. This script is not suitable for the computation of CIs of within-subject effect sizes.

### Data accessibility

The dataset supporting this article has been uploaded as part of the supporting information (S1 File).

## Results

### Matching of participant groups

Prior to the main analysis, we investigated how a range of background variables that were considered as possible confounds (i.e., age, mood, musical training, role of music in life and liking for the piano music heard in the experiment) affected attractiveness and dating desirability ratings of opposite-sex faces in 40 fertile women, 32 infertile women and 40 men. S1 and S2 Tables show Spearman’s rank order correlations between these background variables and ratings of faces in the silent control condition as well as for ratings averaged across the four musical priming conditions. In each of the three participant groups, mostly non-significant small positive and negative correlations emerged for several background variables. Interestingly, several correlations differed considerably in direction and strength across groups: for example, comparing dating desirability ratings of the two female groups revealed that the assumption of homogeneity of regression slopes did not hold for the quietude/disquietude subscale of the MDBF and for liking of piano music. Here, we refrained from employing analysis of covariance to test for the effect of background variables because covariates are known to affect the interpretation of within-subject factors and because it is not clear whether non-significant covariates should be included in the statistical model.

Since it cannot be ruled out that the current background variables contribute cumulatively to ratings of sexual attraction in each group, we decided to keep average values of these variables similar across the three groups to make the groups more comparable. Eight participants in the group of fertile women and another eight men were excluded to create a homogenous sample of participants in terms of age, mood prior to the experiment and musical background variables. Tables 1 and 2 summarize the results of Kruskal-Wallis tests that compared the three groups of the final sample: 32 women in the fertile-phase, 32 women in the infertile phase and 32 men. Importantly, all three groups equally enjoyed listening to the piano music presented in the experiment. The two block and rating orders were counterbalanced across groups (i.e., 16 participants in each condition and group). The three groups neither differed in their wish to have children, χ^2^(2) = 1.22, *p* = .544, nor in their relationship status, χ^2^(2) = 1.92, *p* = .384. Around two thirds of the participants (*n* = 64) expressed a desire to have children. Similarly, around two thirds of the participants were single (*n* = 68), whereas one third indicated to be in a relationship (*n* = 28).

### Effects of music on ratings of sexual attraction

The musical priming paradigm interspersed the trials of interest with distractor trials. Distractor trials (i.e., trials with same-sex faces) were excluded from the main analyses in each participant group. Because women and men were exposed to different sets of opposite-sex faces in the critical trials, separate statistical analyses were conducted for women and men. A set of four orthogonal contrasts within the framework of mixed-design analysis of variance (ANOVA) was used to test our hypotheses regarding ratings of facial attractiveness and dating desirability, respectively. Contrast 1 compared the ratings of the control condition with those of the four musical priming conditions. Contrast 2 compared the two low-arousing with the two high-arousing music conditions. Contrast 3 compared the two pleasant with the two unpleasant music conditions. Contrast 4 tested the interaction between arousal and pleasantness in the music conditions.

**Fig 1:**
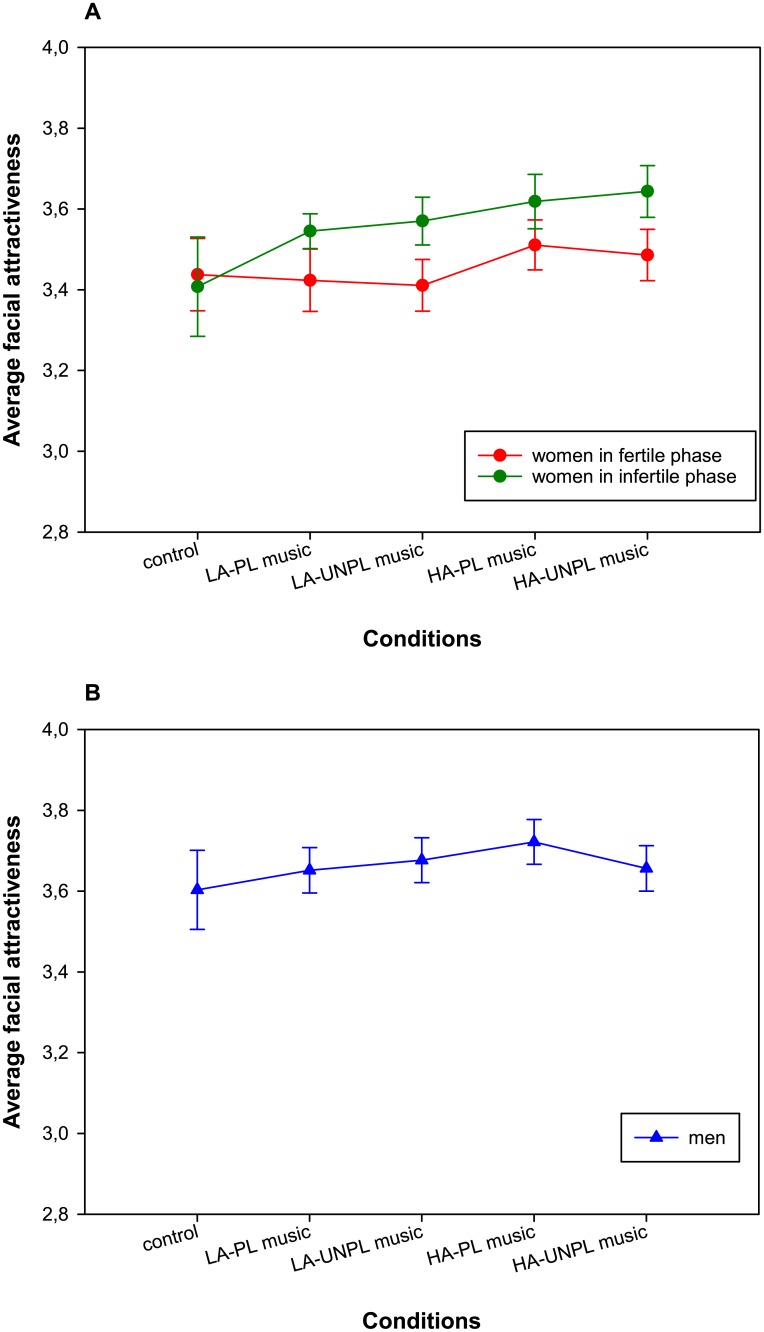
Average facial attractiveness ratings for each experimental condition. (A) Ratings given by women. (B) Ratings given by men. Error bars represent 95% within-subjects confidence intervals. LA-PL = low-arousing pleasant, LA-UNPL = low-arousing unpleasant, HA-PL = high-arousing pleasant, HA-UNPL = high-arousing unpleasant.

To test whether music affected ratings of facial attractiveness in the two groups of women (Fig 1A), a mixed-design ANOVA with condition as a within-subject factor (control and 4 music conditions) and cycle phase (fertile vs. infertile) as a between-subjects factor revealed a significant main effect of condition, *F*(2.8, 171.3) = 4.4, *p* = .007, η_p_^2^ = .07 (medium effect). The condition × cycle phase interaction, *F*(2.8, 171.3) = 1.83, *p* = .149, η_p_^2^ = .03 (small effect), as well as the main effect of cycle phase, *F*(1, 62) = .24, *p* = .629, η_p_^2^ = .004 (no effect), 90% CI [.00; .06], did not reach significance. Next, planned contrasts revealed that attractiveness ratings obtained in the musical priming conditions (*M* = 3.53, *SD* = .87) were higher than in the control condition (*M* = 3.42, *SD* = .81), *F*(1, 62) = 4.93, *p* = .030, η_p_^2^ = .07 (medium effect). Furthermore, high-arousing music (*M* = 3.56, *SD* = .89) led to higher attractiveness ratings than low-arousing music (*M* = 3.49, *SD* = .86), *F*(1, 62) = 12.24, *p* = .001, η_p_^2^ = .17 (large effect). Finally, attractiveness ratings neither differed between the pleasant and unpleasant music conditions, *F*(1, 62) = .02, *p* = .884, η_p_^2^ < .001, nor was there an interaction between arousal and pleasantness in the music conditions, *F*(1, 62) = .02, *p* = .901, η_p_^2^ < .001, suggesting that musical pleasantness did not affect attractiveness ratings. We did not observe any significant interactions with cycle phase for these contrasts (all *p*s > .079).

**Fig 2:**
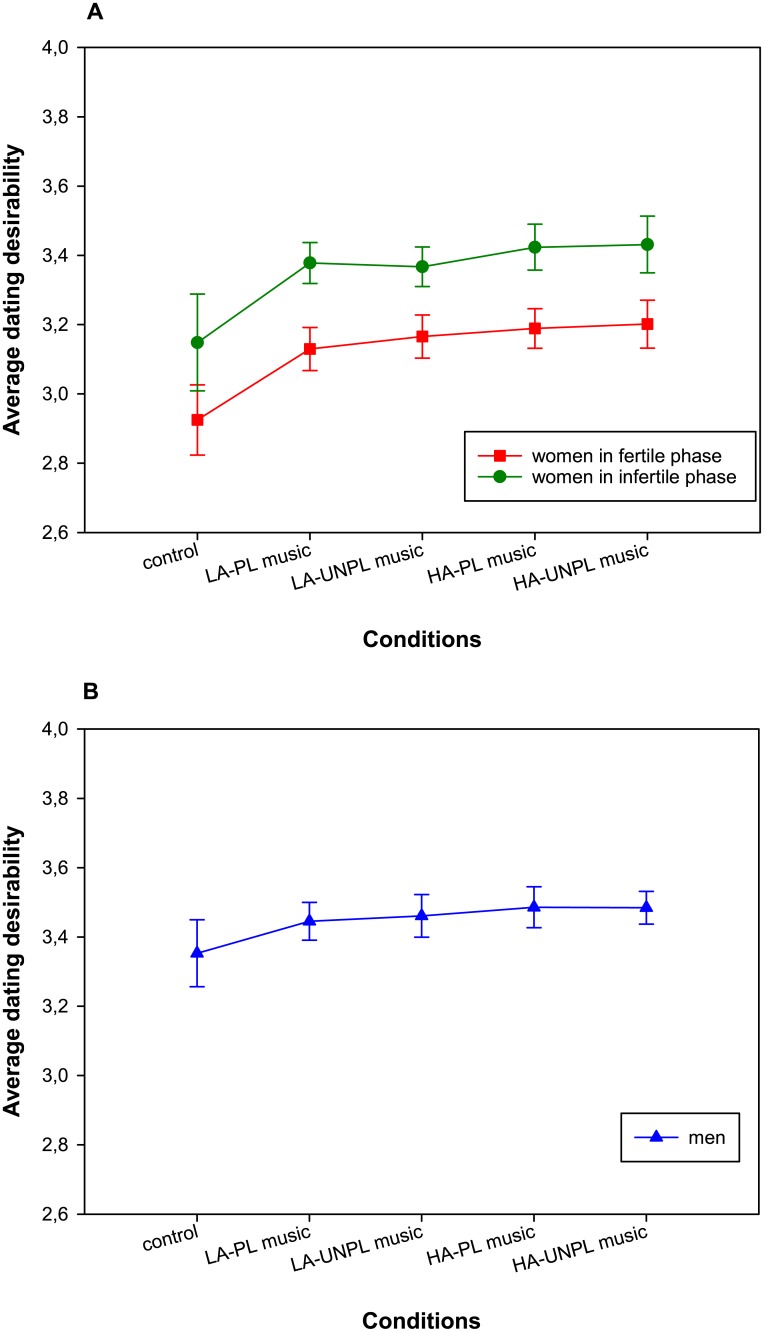
Average dating desirability ratings for each experimental condition. (A) Ratings given by women. (B) Ratings given by men. Error bars represent 95% within-subjects confidence intervals. LA-PL = low-arousing pleasant, LA-UNPL = low-arousing unpleasant, HA-PL = high-arousing pleasant, HA-UNPL = high-arousing unpleasant.

A similar mixed-design ANOVA on ratings of dating desirability showed a large main effect of condition (Fig 2A), *F*(2.4, 147.7) = 13.74, *p* < .001, η_p_^2^ = .18 (large effect). Neither the condition × cycle phase interaction, *F*(2.4, 147.7) = .08, *p* = .989, η_p_^2^ = .001 (no effect), nor the main effect of cycle phase, *F*(1, 62) = .84, *p* = .364, η_p_^2^ = .01 (small effect), 90% CI [.00; .09], were significant. Planned contrasts showed that dating desirability was higher after musical priming (*M* = 3.29, *SD* = 1.03) than in the control condition (*M* = 3.04, *SD* = .90), *F*(1, 62) = 22.15, *p* < .001, η_p_^2^ = .26 (large effect). High-arousing music (*M* = 3.31, *SD* = 1.07) also led to higher dating desirability than low-arousing music (*M* = 3.26, *SD* = 1.02), *F*(1, 62) = 4.74, *p* = .033, η_p_^2^ = .07 (medium effect). Musical pleasantness did not affect dating desirability ratings, *F*(1, 62) = .28, *p* = .596, η_p_^2^ = .005. We did not observe an interaction between arousal and pleasantness in the music conditions, *F*(1, 62) = .002, *p* = .961, η_p_^2^ < .001. We did not observe any significant interactions with phase for these contrasts (all *p*s > .465).

Turning to the group of men, a repeated-measures (RM-) ANOVA with condition as within-subject factor showed that attractiveness ratings did not significantly differ across the five conditions (Fig 1B), *F*(2.6, 80.9) = 1.4, *p* = .239, η_p_^2^ = .04 (small effect). None of the planned contrasts was significant (all *p*s > .088), and the lowest *p*-value was observed for the contrast on the interaction between arousal and pleasantness. Similarly, an RM-ANOVA on ratings of dating desirability showed that ratings in the five conditions were not significantly different (Fig 2B), *F*(2.7, 82.9) = 2.28, *p* = .093, η_p_^2^ = .07 (medium effect). Turning to the contrast analyses, attractiveness ratings were marginally higher in the musical priming conditions (*M* = 3.47, *SD* = 1.06) than in the control condition (*M* = 3.35, *SD* = 1.0), *F*(1, 31) = 3.84, *p* = .059, η_p_^2^ = .11 (medium effect). None of the other three contrasts approached significance (all *p*s > .349). The average within-subject correlation between attractiveness ratings across the five conditions was *r* = .955, the one for dating desirability *r* = .964.

After the experiment, participants were also asked to report their interest in a one-night stand with the persons associated with the faces they considered most attractive in the experiment. A Kruskal-Wallis Test showed that the three groups did not significantly differ in their responses, *H*(2) = 4.7, *p* = .096. Similarly, we assessed participants’ interest in a long-term relationship with the persons with the most attractive faces. We observed a significant difference between the groups, *H*(2) = 6.66, *p* = .036. Follow-up *U*-tests (Bonferroni-corrected) revealed no significant differences between groups, but the data suggested (Table 2) that women in the fertile phase were less interested in a long-term relationship than women in the infertile phase and men.

We also asked participants whether they had identified the purpose of the experiment. The majority of participants was aware that we were interested in the role of music on sexual attraction (*n* = 85), which is not surprising considering that we employed a within-subject design and asked for explicit ratings. When asked to explain the role of music in more detail, the terms musical arousal and pleasantness were never mentioned, which indicates that participants were unaware of the independent variables of interest. However, participants frequently referred to music-induced mood, and 49 out of 92 participants reported that we were interested in mood, a proportion that was similar across experimental groups (men, fertile women, and infertile women), χ^2^(2) = 2.58, *p* = .275.

Last, to increase statistical power by ignoring the influence of background variables, we ran a planned contrast analysis in the framework of mixed-design ANOVA based on the initial sample of 40 fertile women, 32 infertile women and 40 men. In general, we found a similar pattern of results for ratings of attractiveness and dating desirability, respectively. However, there were two exceptions: First, in the analysis comprising women, the interaction between condition and phase for the contrast comparing the control and the musical priming conditions was significant for attractiveness ratings, *F*(1, 70) = 4.22, *p* = .044, η_p_^2^ = .06 (medium effect). A closer inspection of this result revealed that this effect was mostly due to the fact that ratings for the low-arousal priming conditions were lower than in the control condition, whereas ratings for the high-arousal priming condition were higher than in the control condition. This tendency (*p* < .10) can already be seen in Fig 1 for the matched samples. The second difference was observed regarding the analysis of dating desirability in men, in which the contrast between the silent control condition and the musical priming conditions was significant, *F*(1, 39) = 5.17, *p* = .029, η_p_^2^ = .12 (medium effect), showing that ratings of dating desirability were higher under musical priming. A trend for this effect (*p* < .10) was present in the restricted sample of 32 men.

In the analysis of women’s ratings, we also had a closer look at the between-subjects factor (cycle phase) and its change in effect size in comparison to the restricted but matched sample. For attractiveness ratings, cycle phase was not significant, *F*(1, 70) = 0.03, *p* = .863, η_p_^2^ = .00 (no effect), 90% CI [.00; .02], which was also the case for dating desirability ratings, *F*(1, 70) = 0.41, *p* = .526, η_p_^2^ = .01 (small effect), 90% CI [.00; .07].

## Discussion

In a highly controlled experimental setting, we examined the role of music in sexual attraction by applying a crossmodal priming paradigm, using opposite-sex faces as targets. Our finding that music can influence the perception of facial attractiveness among women is in line with observations by May and Hamilton [21] However, in contrast to this study, we employed a single musical style and considered musical arousal as well as pleasantness, enabling us to suggest that misattribution of musical arousal may be the underlying mechanism. Indeed, we observed the largest effects for pleasant and unpleasant high-arousing music compared to a silent control condition, a finding which adds to a considerable body of research showing excitation transfer in the domain of sexual attraction for a wide range of arousal-inducing stimuli and events, for a review see [29]. More specifically, our results indicate that both positively- and negatively-valenced musical arousal can lead to positive effects on sexual attraction, thus pointing towards a general arousal mechanism that may affect social behaviour in a mating context [31].

Our findings have also implications for crossmodal research involving musical and visual stimuli. Besides misattribution of musical arousal as a candidate mechanism for increased ratings of facial attractiveness, future research may study whether music may specifically alter the perception of traits such as weight, color, averageness, symmetry, masculinity and good behaviour as well as personality [18]. Furthermore, our results relate to crossmodal emotion research [47]. Musical emotions have a clear impact on the perception of visual emotions, and the current results are in line with our previous finding that music-induced arousal influences subjective arousal responses—but not pleasantness responses—elicited by affective environmental scenes [26].

Furthermore, we were interested in whether gender and menstrual cycle phase may act as moderators. Our results clearly demonstrate that music-induced arousal can significantly prime dating desirability, and to a lesser degree, the perceived sexual attractiveness of male faces in women taking no hormonal contraceptives. In light of Darwin’s theory [5], these results generally support the idea that the experience of music may play a role in women’s social behaviour in a mating context, and especially that high-arousing (i.e., more complex) music affects the perception of male facial attractiveness and dating desirability. However, contrary to Darwin’s theory, the effects of music were not significant among male participants matched to the female groups for a range of background variables, although there were indications for an effect of music on dating desirability in the unmatched male sample. One possible interpretation is that men generally use courtship displays to attract women, and not vice versa, as implied by previous studies focusing primarily on how women are attracted by men’s musical engagement or abilities [9, 10, 11]. This interpretation is also supported by a recent study that focused on the effect of creative displays (ability to write short-story extracts in relation to a painting and divergent thinking) on judgements of facial attractiveness in men and women, demonstrating how indicators of intelligence, such as creativity, can impact on social judgements during mate choice [48]. Our results further show that in the case of music, the effects of music can be observed without establishing a conscious link between musical ability and the facial cue in the experimental design. Future research may also focus on the systematic study of music’s effect on attraction to same-sex faces, particularly in heterosexual women [49]. The current research design was limited both by the number of available musical primes and by practical concerns relating to the duration of the experiment.

Contrary to the ovulatory shift hypothesis [37], we did not observe significant differences between ratings by women in the fertile versus infertile phase ([12], but see [9] who reported a large effect). We partly attribute this discrepancy with Charlton [9] to the difference in experimental designs, sample size and the manner of determining fertility. Whereas Charlton combined musical stimuli with the evoked concept of a fictitious composer who created these stimuli in a large online study, we were primarily interested in the possible link between music and face processing regarding sexual attraction. In our paradigm, we did not explicitly state that the musical excerpt has been played or created by the person depicted on the photograph. Furthermore, we did not study differences between music’s effects on short- and long-term mating strategies [36], an issue that remains to be tackled in the future.

Current issues pertaining to the ovulatory shift hypothesis [35] may limit the interpretation of our results. First, it is still debated how fertility can effectively be determined and how the chosen method relates to sample size [40]. Second, we think that the debate on statistical power and sample size in this context frequently ignores the value of matching groups on influential background variables and the quality of the stimuli used in an experiment. Third, large sample sizes may be necessary to detect small effects of fertility on visual attractiveness ratings among women, as has been shown recently by a study on visual attributes of male attractiveness [50]. However, it is not always possible to recruit such large samples if certain restrictions, such as low musical training and no hormonal contraceptive use, need to be considered, especially not in a cohort of university students. Fourth, when new paradigms are applied, as in the present case, it is difficult to predict an effect size in advance. Taken together, we regard it as a challenging task to include menstrual cycle phase as a factor at the moment.

Last but not least, we hope that our study contributes to the discussion on mechanisms that may explain how musical ability affects sexual attraction. The perceived musical ability of one single performer or musician may directly act as an honest signal for a potential mate, linking performance skills with mate choice [9]. Indeed, anthropological evidence suggests that in several tribes around the world playing an instrument is used to attract a mate [13, 14]. However, it is still unclear whether conscious appreciation of the musical skills of a potential partner leads to mate choice. Underlying affective, less cognitive, mechanisms may as well lead to attraction, or cognitive and affective mechanisms may interact with each other. This is not a trivial hypothesis since the nature of sexual culture differs largely in tribal societies [51, 52, 53], and sexual practices may take different forms even within one tribe and depend on age and relationship status, such as in the Maasai tradition [53]. For example, it is common in some tribal cultures to have several sexual partners at the same time within the same group, chosen after a ritual, but also to have sexual relations with members from other tribes. Moreover, it has been frequently suggested that sexual relations and customs cannot be discussed in isolation because they are tightly interwoven with politics and economics [54, 55] The diversity of sexual traditions and the various forms of rituals in which music is performed make it difficult to pin down the exact mechanisms through which music may have affected sexual attraction.

In conclusion, this research project complements ongoing genetic [15] and neurophysiological [4] research on the biological roots of music as well as research on interpersonal attraction by implying that music reinforces women’s sexual attraction towards men through crossmodal arousal induction. Musical pleasantness as well as menstrual cycle phase appear to be less important factors. Our findings thus clarify how music affects facial attraction and prompt further study of the mechanisms underlying music’s powerful role in parental care or social group cohesion, which together with the sexual selection theory of music may explain the biological roots of musical behaviour [4].

## Supporting information

S1 Table Spearman’s rank order correlations between average attractiveness ratings and age, mood, and musical background variables for three groups of participants. (PDF)

S2 Table Spearman’s rank order correlations between average dating desirability ratings and age, mood, and musical background variables for three groups of participants. (PDF)

S1 File Data file containing experimental data and background variables of individual participants. (XLSX)
