# Supplementary material for: Misattribution of musical arousal increases sexual attraction towards opposite-sex faces in females
Source: PLoS One. 2017 Sep 11;12(9):e0183531. doi: 10.1371/journal.pone.0183531 (PMC5593195; doi:10.1371/journal.pone.0183531)
Supplement: S1 Table — (PDF) [file pone.0183531.s001.pdf]

S1 Table.

Spearman's rank order correlations between average attractiveness ratings and age, mood, and musical background variables for three groups of participants.

Group	Condition	Age	Mood pos.-neg.	Alertness/ fatigue	Quietude/ Disquietude	Yrs. musical training	Role of music in life	Liking of piano solo music
Fertile women								
Df = 38	control	-.003	-.091	-.026	-.251	.281	-.280	.065
	music	.087	-.017	.012	-.199	.251	-.277	.140
Infertile women								
Df = 30	control	-.034	.171	.255	.168	-.128	.023	.168
	music	.027	.023	.229	.123	.080	-.015	.258
Men								
Df = 38	control	-.258	.107	.125	.101	-.250	.021	.108
	music	-.247	.117	.083	-.004	-.263	.025	.021

Note. Correlations of notable strength are highlighted in grey. Df = degrees of freedom. Control = ratings for silent control condition; Music = ratings for averaged musical priming conditions.
