# Supplementary material for: Misattribution of musical arousal increases sexual attraction towards opposite-sex faces in females
Source: PLoS One. 2017 Sep 11;12(9):e0183531. doi: 10.1371/journal.pone.0183531 (PMC5593195; doi:10.1371/journal.pone.0183531)
Supplement: S2 Table — (PDF) [file pone.0183531.s002.pdf]

S2 Table.

Spearman's rank order correlations between average dating desirability ratings and age, mood, and musical background variables for three groups of participants.

Group	Condition	Age	Mood pos.-neg.	Alertness/ fatigue	Quietude/ Disquietude	Yrs. musical training	Role of music in life	Liking of piano solo music
Fertile women								
Df = 38	control	-.187	-.004	-.123	-.328*	.350*	-.139	-.060
	music	-.093	.171	-.030	-.265	.291	-.236	-.086
Infertile women								
Df = 30	control	-.003	.204	.239	.303	.055	.065	.354*
	music	-.072	.017	.282	.139	.138	-.039	.255
Men								
Df = 38	control	-.278	.132	.098	.026	-.278	.074	.218
	music	-.275	.110	.061	-.053	-.299	.032	.098

Note. Correlations of notable strength are highlighted in grey. Df = degrees of freedom. Control = ratings for silent control condition; Music = ratings for averaged musical priming conditions. * $p < .05$.
